# Molecular and immune characteristics of neuroendocrine bladder carcinoma—Implications for diagnosis, prognosis, and therapy: A review

**DOI:** 10.17305/bb.2025.13151

**Published:** 2025-10-09

**Authors:** Tianxiang Zhang, Xi Zhang, Lei Qian, Chunjiang Hu, Jianxing Li

**Affiliations:** 1 Department of Urology, Tsinghua University Affiliated Beijing Tsinghua Changgung Hospital, Beijing, China; 2 School of Clinical Medicine, Tsinghua University, Beijing, China; 3 State Key Laboratory of Systems Medicine for Cancer, Department of Urology, Shanghai Cancer Institute, Ren Ji Hospital, Shanghai Jiao Tong University School of Medicine, Shanghai, China

**Keywords:** Neuroendocrine bladder carcinoma, driver genes, immune microenvironment, molecular features, immune checkpoint inhibitors

## Abstract

Neuroendocrine bladder carcinoma (NEBC) is a rare but highly aggressive histologic subtype of bladder cancer with poor prognosis, often driven by delayed diagnosis and limited therapeutic options; despite widespread use of next-generation sequencing, its cellular origin remains unclear and controversial. We aimed to synthesize up-to-date molecular and immune features of NEBC and translate them into practical guidance for diagnosis and treatment. We performed a narrative review of English-language studies indexed in PubMed and Web of Science (January 2000–August 2025) using predefined keywords, integrating genomic, transcriptomic, immunohistochemical, and clinical outcome data. Key findings indicate frequent co-occurrence and probable common clonal origin with urothelial bladder carcinoma, with hallmark *TP53* and *RB1* alterations, prevalent APOBEC-driven mutagenesis, and recurrent *TERT* promoter mutations; tumor mutation burden is heterogeneous but can be high. Despite this, NEBC commonly exhibits an immune-cold or immune-excluded microenvironment characterized by low PD-L1 expression and T-cell dysfunction, which may blunt responses to immune checkpoint inhibitor (ICI) monotherapy. Diagnostic practice still relies on morphology supported by immunohistochemistry (synaptophysin, chromogranin A, CD56, GATA3), with emerging tools such as INSM1 and a decision-tree model using synaptophysin, CD117, and GATA3 that improve accuracy. Therapeutically, neoadjuvant chemotherapy (NAC)—most commonly EP or IA—followed by radical cystectomy improves outcomes compared with initial cystectomy alone, while metastatic disease is typically managed with EP chemotherapy and radiotherapy with limited durability. Early data support immunotherapy, particularly ICIs, and suggest potential benefit from chemoimmunotherapy; a prospective trial of neoadjuvant anti-PD-L1 plus EP is underway, and antibody-drug conjugates and bladder-sparing multimodality strategies are emerging. In conclusion, comprehensive molecular and immune characterization is critical to refine diagnosis, optimize patient selection, and accelerate prospective trials that evaluate NAC, chemoimmunotherapy, and targeted approaches in NEBC.

## Introduction

Bladder cancer (BC) is the most prevalent malignant tumor of the urinary system, accounting for over 570,000 new cases and more than 210,000 deaths globally each year [1, 2]. BC is a heterogeneous disease characterized by various histological subtypes, including urothelial carcinoma, adenocarcinoma, squamous cell carcinoma, and neuroendocrine carcinoma [3]. Neuroendocrine tumors can develop in several anatomical sites, such as the sympathetic nervous system, adrenal gland, lung, pancreas, bladder, and prostate [4]. Regardless of their origin, neuroendocrine tumors consist of neuroendocrine cells that secrete bioactive substances and proteins, including somatostatin, insulin, gastrin, serotonin, and synaptophysin [5]. Histologically, neuroendocrine bladder carcinoma (NEBC) is classified as small cell carcinoma, large cell carcinoma, or mixed neuroendocrine carcinoma [6, 7]. Although NEBC is rare, representing less than 2% of BC diagnoses, it is an exceedingly aggressive disease [8]. NEBC is often diagnosed at an advanced stage, associated with a high metastatic potential and a 5-year survival rate of less than 10% [9, 10]. Consequently, early diagnosis and multimodal treatment strategies are crucial for managing NEBC [11–13]. Currently, NEBC diagnosis lacks a gold standard, relying primarily on morphological findings supplemented by immunohistochemical stains [7]; imaging serves only as an adjunctive tool and is not essential for definitive diagnosis [14].

Therapeutically, clinical guidelines for NEBC predominantly draw from management strategies for urothelial carcinoma and other neuroendocrine carcinomas (e.g., small cell lung cancer), supported by limited high-quality evidence [15]. The scarcity of NEBC cases in clinical trials further hampers the development of innovative therapeutic approaches [10]. In recent years, immunotherapy has emerged as a significant advancement in the treatment of urothelial cancer and other neuroendocrine carcinomas [16]. Among these therapies, immune checkpoint inhibitors (ICIs), particularly those targeting the PD-L1/PD-1 pathway, enhance T-cell-mediated tumor cytotoxicity, thereby exerting anti-tumor effects [17]. Our recent study has also demonstrated the superior efficacy of combined chemoimmunotherapy in NEBC [18]. However, a comprehensive understanding of the molecular and immune mechanisms underlying NEBC remains incomplete.

This review aims to synthesize current knowledge regarding the molecular and immune landscape of NEBC and provide translational insights for implementing these findings in personalized clinical management.

## Literature search strategy

This literature review explores recent advancements in the molecular and immune characteristics of NEBC and their implications for diagnosis and treatment. We conducted a search in the PubMed and Web of Science databases for published English-language articles from January 2000 to August 2025. The search strategy employed a combination of keywords, including “neuroendocrine bladder carcinoma,” “bladder cancer,” “immune microenvironment,” “molecular features,” “immune checkpoint inhibitors,” and “neuroendocrine cancer.” Boolean operators (AND/OR) were utilized to refine search results.

## The origin of NEBC

### Common clonal origin hypothesis

Emerging evidence suggests that NEBC and urothelial bladder carcinoma (UBC) share a common cellular origin. In 2005, Cheng et al. [19] first proposed this common clonal origin of small cell carcinoma of the bladder (SCBC) and UBC at the molecular genetic level. They identified similar patterns of allelic imbalance and X-chromosome inactivation between SCBC and coexisting UBC, indicating that these tumors may arise from undifferentiated, multipotent progenitor cells within the urothelium [7, 19]. The heterogeneity of NEBC presents significant challenges to the accurate immunohistochemical identification of large cell NEBC [20]. NEBC and UBC are frequently observed together during histopathological examinations [7]. Approximately 40% of SCBC cases have been documented to exhibit mixed histological components of small cell and urothelial carcinomas [13]. Furthermore, NEBC and UBC often share similar somatic mutations within the same lesion, suggesting a clonal relationship between the two cancer types [19, 21]. Studies utilizing comparative genomic hybridization, next-generation sequencing, and immunohistochemistry have indicated that urothelial carcinoma may transform into NEBC through the accumulation of genetic mutations [22, 23]. Shen et al. [24] later demonstrated that the genomic profiles of NEBC are comparable to those of conventional UBC. Notably, both NEBC and UBC exhibit similar carcinogenic pathways driven by age-related and *APOBEC*-mediated mutational processes. By comparing genomic data from tumor samples of 87 SCBC cases to those of 303 high-grade UBC and 149 small cell lung cancers, Chang et al. [22] identified a similar histology-specific mutational pattern of somatic *RB1* and *TP53* driver mutations in SCBC and UBC, which were absent in small cell lung cancers. A comparative analysis of 25 BC cases coexisting with SCBC and non-small-cell phenotypes in the urothelium revealed an identical somatic mutation in the *TERT* promoter across both components [21]. Experimentally, Wang et al. [25] constructed a patient-derived xenograft model demonstrating that genetically engineered urothelial cells can give rise to mixed histological subtypes of NEBC and UBC. Additionally, another study suggested that *miR-145* could induce a stem cell-like phenotype in urothelial carcinoma cells, promoting their differentiation into neuroendocrine cells by inhibiting syndecan-1 [26]. A case report from Robert-Bosch Hospital further illustrated that an invasive tumor developed within classical urothelial carcinoma in situ, comprising a mixed tumor of urothelial carcinoma in situ, NEBC, and an adenocarcinomatous component, with concurrent upregulation of *p53* and strong cytoplasmic and membranous β-catenin staining [27]. Collectively, this evidence supports the hypothesis of a common origin between NEBC and UBC. However, further preclinical experimental models, particularly organoid models, warrant exploration to validate this hypothesis, as seen in studies involving small cell lung carcinoma and neuroendocrine prostate cancer [28–32].

### Other hypotheses

While considerably less common, alternative theories regarding the origins of NEBC have been proposed. A study utilizing lineage tracing in a murine model of BC indicated that fundamental differences in the cell of origin may account for variations in clinical course, prognosis, and histological morphology, potentially explaining the distinctions between NEBC and UBC [33]. Additionally, a case study by Olivieri et al. found that UBC expressed cytokeratin but lacked synaptophysin expression, while NEBC co-expressed both markers. This led them to propose that NEBC may originate from the neuroendocrine system rather than from urothelial cells [34].

## Molecular characteristics of NEBC

Emerging evidence suggests that BC ranks among the most frequently mutated human tumors, following lung and skin cancers in mutation frequency [35, 36]. This section examines the critical molecular alterations in NEBC and their potential implications.

### TP53 and RB1

The inactivation of *TP53* and *RB1* serves as a significant biomarker for NEBC [37]. Alterations in these genes are present in nearly 80% of poorly differentiated neuroendocrine carcinomas [38]. Dysfunction of *TP53* and *RB1* is associated with histological progression to neuroendocrine carcinoma in lung and prostate cancers [39–42]. Recent studies have reported high mutation frequencies of *TP53* and *RB1* in NEBC. For instance, a study involving 61 SCBC patients indicated mutation frequencies of up to 90% for both genes [22]. Additionally, 80% of SCBC patients exhibited double mutations in *TP53* and *RB1*. Similar findings emerged from research conducted by the Johns Hopkins Greenberg Bladder Cancer Institute, which documented mutation frequencies of 92% for *TP53*, 75% for *RB1*, and 72% for concurrent *TP53*/*RB1* mutations in 132 SCBC patients [43]. Another study identified genetic alterations in *TP53* and *RB1* linked to reduced responsiveness to targeted therapies [24]. Notably, integrative analyses of muscle-invasive BC identified a neuronal subtype where 10 of 20 (50%) tissues displayed either both *RB1* and *TP53* alterations or *E2F3* amplification [37]. Furthermore, 17 out of 20 (85%) tumor samples harbored somatic mutations in the p53/cell-cycle signaling pathway. Interestingly, in cases of UBC, inactivating mutations in *TP53* and *RB1* were reported in 12% of cases, suggesting these mutations may be sufficient, but not necessary, for the transformation into NEBC [44].

### APOBEC

The apolipoprotein B mRNA editing enzyme, catalytic polypeptide (*APOBEC*) family comprises a group of cytosine deaminases [45]. Analyses of The Cancer Genome Atlas (TCGA) data indicate that APOBEC-mediated mutagenesis significantly contributes to BC carcinogenesis [44]. Conversely, another study integrating whole-exome sequencing, next-generation sequencing, and transcriptome analysis suggested that high APOBEC activity correlates with favorable prognosis, immune activation, and response to immune-checkpoint blockade in BC [46]. Specifically, in NEBC, *APOBEC*-driven mutational events occur in 95% of SCBC patients, potentially resulting in a high mutation burden [22]. Our previous study identified a prevalent APOBEC-mediated subtype characterized by distinct mutational signatures in NEBC patients [18, 24, 47]. Additionally, Robertson et al. identified a neuronal subtype in muscle-invasive BC associated with genes mutated alongside APOBEC activity [37, 47].

### TERT promoter

Mutations in the TERT promoter represent a frequent molecular characteristic of NEBC. One study detected *TERT* promoter mutations in 55% (29 of 53) of SCBC cases [21]. Another study reported that 100% (10 of 10) of NEBC cases exhibited the *TERT* promoter *C228T* mutation [23]. Notably, SCCs from other cancer types, including prostate, lung, cervix, esophagus, and skin, did not harbor TERT promoter mutations, highlighting its potential as a diagnostic biomarker [23, 48]. Furthermore, the common allele rs2853669 within the TERT promoter mutation was associated with reduced overall survival and an elevated risk of tumor recurrence in BC [49, 50].

## Immune features of NEBC

The bladder urothelium is continuously exposed to urinary carcinogens, such as tobacco-derived compounds, microbiota, and aromatic hydrocarbons. This ongoing exposure renders BC a highly immunogenic disease, often characterized by a high somatic mutation rate and an abundance of tumor neoantigens [51, 52]. Consequently, BC is particularly amenable to immunotherapy, resulting in the approval of multiple ICIs for clinical use [53]. However, the efficacy of immunotherapy remains limited in a subset of BC patients. Urgent investigation is warranted to comprehensively characterize the immune microenvironment of NEBC, identify responsive patient subgroups, and develop optimized therapeutic strategies.

**Figure 1.**
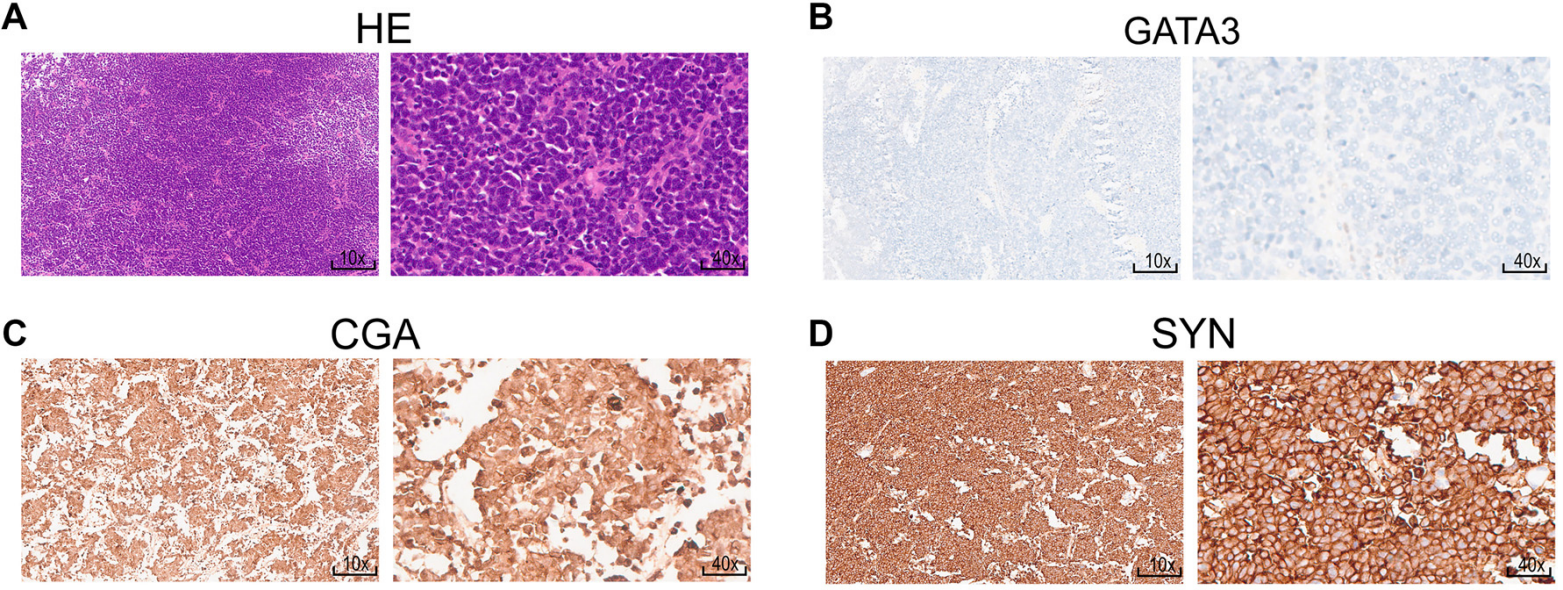
**Hematoxylin–eosin and immunohistochemistry staining of representative markers for the diagnosis of NEBC.** (A) Hematoxylin–eosin staining of NEBC tissues. Immunohistochemistry staining of GATA3 (B), CGA (C) and SYN (D) in NEBC samples. 10× corresponds to 200 um, and 40× corresponds to 50 um. The images and stains were done following the approval from the Ethics Committee of Ren Ji Hospital (approval code: KY2022-038-B). The following primary antibodies were used: Anti-CGA (Proteintech, catalog no. 10529-1-AP, 1:500), anti-GATA3 (Proteintech, catalog no. 66400-1-Ig, 1:100), and anti-SYN (Proteintech, catalog no. 17785-1-AP, 1:1000). Abbreviations: NEBC: Neuroendocrine bladder cancer; GATA3: GATA-binding protein 3; CGA: Chromogranin A; SYN: Synaptophysin.

### Tumor mutation burden (TMB)

TMB, defined as the number of mutations present in a tumor, reflects the level of neoantigens and the likelihood of T-cell recognition [54, 55]. A significant association between TMB and response to immunotherapy has been reported across various tumor types, including non-small cell lung cancer, melanoma, and urothelial carcinoma [56–58]. NEBC exhibits heterogeneous TMB levels. A study of 132 cases of small cell carcinoma of the bladder and upper urinary tract found that 26% of SCBC samples exhibited TMB ≥ 10 mutations/Mb, 3% had TMB ≥ 20 mutations/Mb, and the median TMB value was 6.2 mutations/Mb [43]. Another study involving 17 SCBC patients reported a median TMB of 10.7 (ranging from 1.2 to 41.1) mutations/Mb, significantly higher than observed in other genitourinary tumors [22]. Furthermore, Shen et al. reported an average mutation rate of 12.91 (ranging from 0.6 to 41.4) mutations/Mb in 12 resected genitourinary neuroendocrine neoplasms derived from the bladder [24]. A meta-analysis encompassing 27 tumor types demonstrated that the average response rate positively correlates with the logarithm of TMB [59]. Importantly, BC with a neuroendocrine-like molecular subtype is among the most sensitive tumors to ICIs, suggesting promising therapeutic efficacy for immunotherapy in NEBC [60, 61].

### Immune infiltration

The tumor immune microenvironment—particularly the infiltration of CD8+ T cells associated with anti-tumor effects—enables the classification of NEBC into two (immune-cold and immune-hot) or three primary immunophenotypes (immune-inflamed, immune-excluded, and immune-desert) [62–64]. Despite high TMB, NEBC predominantly exhibits an immune-cold phenotype. Based on transcriptome sequencing of 24 SCBC cases and 51 UBC cases, Jean Hoffman-Censits et al. demonstrated that the expression of T-cell-related markers and inflammatory signaling pathways was suppressed in SCBC [43]. Another study comparing potential predictors between 12 SCBC and 69 UBC by immunohistochemistry concluded that SCBC primarily exhibited an immune-excluded subtype, which is characterized by the absence of PD-L1 expression and few tumor-infiltrating lymphocytes in the center of the cancer [65]. Similar findings were reported in small cell lung cancer research [66]. Chan et al. [67] identified an immunosuppressive tumor microenvironment in small cell lung cancer through single-cell sequencing, characterized by CD8+ T cell exhaustion. The distinct immune-excluded phenotype of NEBC may compromise the therapeutic efficacy of monotherapy with ICIs.

## Clinical management of NEBC

### Diagnosis

NEBC is characterized by an aggressive clinical course, typically presenting at an advanced stage with a high propensity for metastasis, resulting in a low overall 5-year survival rate of 8%–25%. Key negative prognostic factors include age over 65 years, advanced TNM stage, and metastatic disease at diagnosis, with tumor stage being the most significant predictor [3]. The diagnosis of NEBC poses considerable challenges for both pathologists and clinicians [68]. Currently, clinical diagnosis primarily relies on pathological morphology and immunohistochemistry [69]. Small cell neuroendocrine carcinoma is defined histologically by features such as sheets and nests of small cells, scant cytoplasm, speckled nuclei, and indistinct nucleoli [70, 71]. In the urinary bladder, NEBC often presents as a mixed component of SCBC and non-SCBC [12, 72]. Diagnosing large cell NEBC is considered more challenging than that of SCBC due to its morphologic characteristics. Large cell NEBC and mixed NEBC cases frequently exhibit enlarged nuclei, which may lead to misdiagnosis as high-grade urothelial carcinoma and delays in appropriate clinical intervention [6]. Distinguishing features between SCBC and large cell NEBC include larger tumor cells, a lower nuclear-to-cytoplasmic ratio, and prominent nucleoli in large cell NEBC [73]. Traditional neuroendocrine markers for NEBC, such as synaptophysin, chromogranin A (CGA), and CD56, have their limitations. For instance, CGA demonstrates a lack of sensitivity [7, 74], while CD56 exhibits high sensitivity but low specificity [75]. A combination of morphologic features and traditional immunohistochemical markers, including GATA3, CGA, and synaptophysin, is widely employed for NEBC diagnosis in clinical practice (Figure 1) [70, 74]. However, immunohistochemical staining may be focal or weak, and often only a few markers yield positive results. Thus, histomorphology alone may suffice for diagnosis, as neuroendocrine markers can be negative in 10% of cases [76]. There is an urgent need for novel diagnostic methods to enhance accuracy for NEBC. Kim et al. [70] developed a decision tree model based on synaptophysin, CD117, and GATA3, achieving 98.4% accuracy in identifying neuroendocrine differentiation in NEBC. Additionally, insulinoma-associated protein 1 (INSM1) has emerged as a promising diagnostic biomarker for neuroendocrine carcinomas across various anatomical sites, including the uterine cervix, pancreas, prostate, thoracic cavity, and head and neck, demonstrating both high sensitivity and specificity [77–80]. After evaluating INSM1 staining in NEBC, a study demonstrated that INSM1 was positive in 87% (28 of 32) of cases, highlighting its potential as a diagnostic tool for NEBC [81]. Moreover, neuronal markers identified through RNA sequencing or immunohistochemistry can also be utilized to define the neuroendocrine subtype in urothelial carcinoma, as these tumors may not exhibit the classic morphologic features of neuroendocrine neoplasms [37, 82].

**Table 1:** Representative studies exhibit the survival outcomes of NAC in NEBC patients

**Study (first author)**	**Treatment**	**Number of patients**	**Regimen**	Downstaging rate*	**Median survival months**	**5-year survival rate**	
Siefker-Radtke [85]	RC only	25	NA	–	23 (CSS)	36%	
	NAC+RC	21	IA or EP	57%	Not reached	78%	
Siefker-Radtke [86]	NAC+RC	18	IA or EP	78%	58 (OS)	–	
Lynch [87]	RC only	47	NA	–	18.3 (OS)	20%	
	NAC+RC	48	IA or EP etc.	62%	159.5 (OS)	79%	
Vetterlein [88]	RC only	144	NA	–	17.3 (OS)	–	
	NAC+RC	125	cisplatin-based	15.2% (pCR)	34.7 (OS)	–	
Alhalabi [89]	RC only	38	NA	21.1%	20.6 (OS)	22%	
	NAC+RC	141	EP or IA etc.	49.5%	86.1 (OS)	57%	
Bakaloudi [90]	NAC+RC	29	EP or CE or GC	–	46 (OS)	41%	

*Downstaging rate refers to pathologic stage ≤ pT1N0 proportion at cystectomy; “–” represents unclear data. The study from Vetterlein is a study of a broader variant-histology cohort, while the other five studies are about NEBC-only cohorts. Abbreviations: RC: Radical cystectomy; NAC: Neoadjuvant chemotherapy; IA: Ifosfamide plus doxorubicin; EP: Etoposide and cisplatin; CE: Carboplatin and etoposide; GC: Gemcitabine and cisplatin; CSS: Cancer specific survival; OS: Overall survival; pCR: Pathologic complete response.

**Figure 2.**
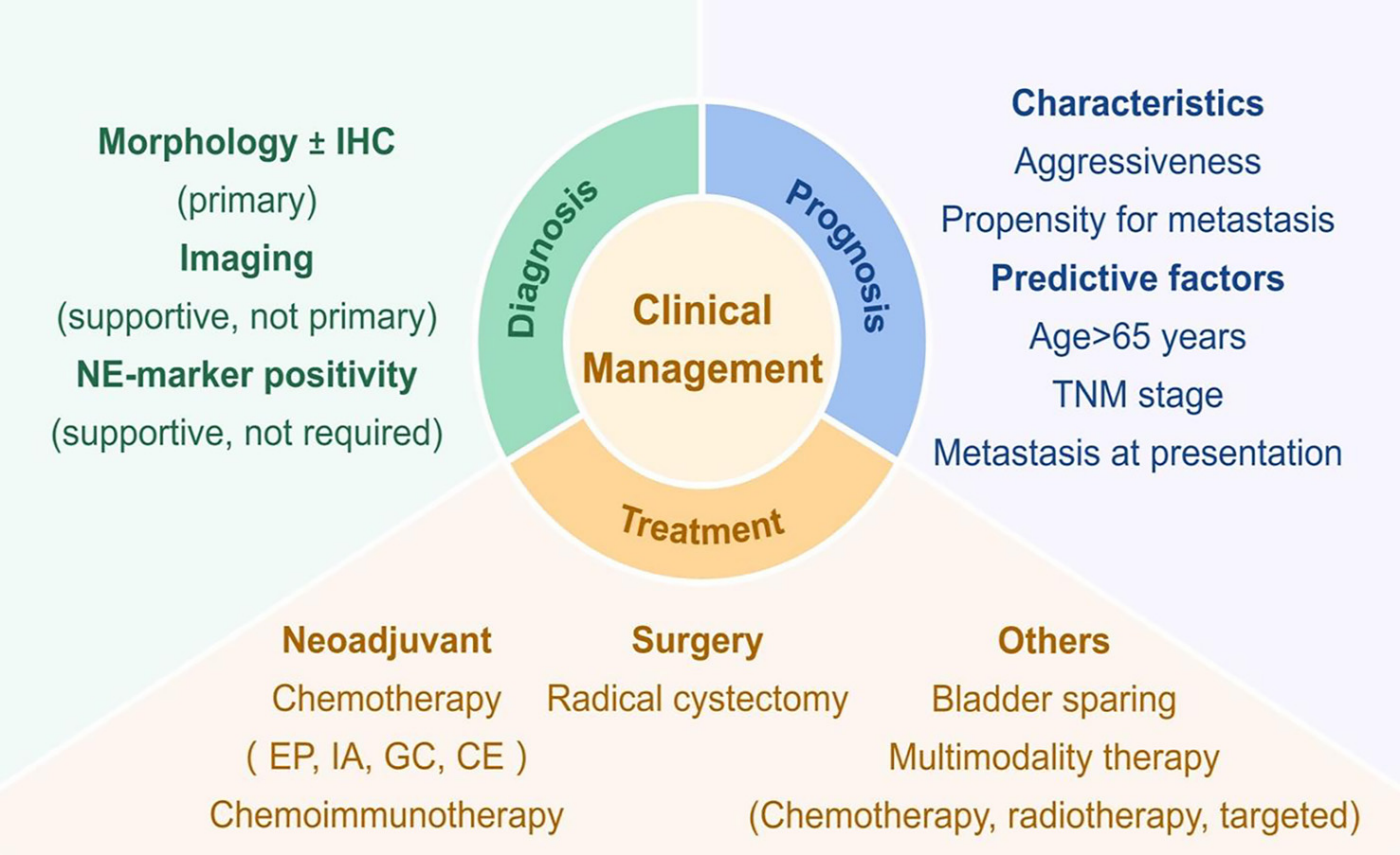
**Overview of NEBC management: Diagnosis, prognosis, and treatment.** Abbreviations: IHC: Immunohistochemistry; NE: Neuroendocrine; EP: Etoposide and cisplatin; IA: Ifosfamide plus doxorubicin; GC: Gemcitabine and cisplatin; CE: Carboplatin and etoposide; TNM: Tumor-node-metastasis.

### Treatment

The sensitivity of neoadjuvant chemotherapy (NAC) in NEBC has been confirmed in reports from various institutions [13, 83]. Recent studies have demonstrated significantly improved outcomes in SCBC patients receiving NAC, with 5-year cancer-specific survival rates increasing from 38% to 78% [84]. We summarized prior studies of large NEBC patient cohorts (Table 1), we found that IA (ifosfamide and doxorubicin) or EP (etoposide and cisplatin)-based NAC combined with radical resection resulted in significantly better survival outcomes than those observed in patients who did not receive NAC [84–89]. However, for patients with metastatic NEBC unsuitable for surgery, current treatment options are limited to EP chemotherapy and radiotherapy for metastatic lesions [37, 90]. Although these therapies achieve a relatively favorable response rate, limited progression-free survival and drug resistance remain prevalent [87, 91]. Given the high immunogenic potential of BC, immunotherapy, particularly ICIs, represents a promising therapeutic strategy for various BC subtypes, including NEBC [92]. A study reported that a recurrent metastatic NEBC patient responded favorably to pembrolizumab therapy in the sixth-line setting, with minimal drug toxicity [93]. Conversely, another retrospective study indicated that BC patients with neuroendocrine features exhibited shorter overall survival following ICI therapy compared to those with pure urothelial carcinoma [94]. Furthermore, our real-world experiences with off-label ICI use suggest that chemoimmunotherapy—a combination of chemotherapy and immunotherapy—might provide a therapeutic advantage for certain NEBC patients compared to chemotherapy alone [18]. Building on these promising preliminary findings, we have initiated a prospective clinical trial (ClinicalTrials.gov identifier: NCT06091124; Registration date: November 16, 2023; Registry: Ren Ji Hospital) to formally assess the efficacy and safety of neoadjuvant adebrelimab (anti-PD-L1) plus EP in patients with NEBC. Additionally, novel targeted therapies, particularly antibody-drug conjugates such as rovalpituzumab tesirine and sacituzumab govitecan, have shown preliminary efficacy in small cell lung cancer and NEBC [94–96]. Furthermore, innovative bladder-preserving approaches have been widely applied in the treatment of muscle-invasive BC. For instance, our preliminary findings suggest the safety and efficacy of combining disitamab vedotin with toripalimab and radiotherapy as a multimodal organ-sparing strategy for muscle-invasive BC [97]. As these approaches mature, their future application in bladder-preserving therapy for non-metastatic NEBC appears promising. Overall, compared to other neuroendocrine carcinomas, therapeutic options for NEBC remain limited. Additional novel therapies should be evaluated through prospective clinical trials involving NEBC patients.

## Conclusion

Given its aggressive nature and unfavorable prognosis, NEBC requires prompt clinical intervention (Figure 2). The origin of NEBC remains unclear and controversial, necessitating further research to elucidate this phenomenon. Notably, the coexistence of high TMB and immune exclusion in the NEBC microenvironment offers novel insights for guiding immunotherapy. A comprehensive understanding of the molecular characteristics and an increased number of well-designed clinical trials are essential for addressing this aggressive subtype of BC.

## Data Availability

No datasets were generated or analysed during the current study.
